# Racial/Ethnic differences in receipt of naloxone distributed by opioid overdose prevention programs in New York City

**DOI:** 10.21203/rs.3.rs-2934002/v1

**Published:** 2023-05-17

**Authors:** Shayla Nolen, Andrew J. Trinidad, Ashly E. Jordan, Traci C. Green, Ali Jalali, Sean M. Murphy, Xiao Zang, Brandon D.L. Marshall, Bruce R. Schackman

**Affiliations:** Brown University School of Public Health; New York City Department of Health and Mental Hygiene; New York City Department of Health and Mental Hygiene; Warren Alpert School of Medicine of Brown University; Weill Cornell Medical College; Weill Cornell Medical College; University of Minnesota; Brown University School of Public Health; Weill Cornell Medical College

**Keywords:** Naloxone, racial/ethnic disparities, opioid overdose prevention programs, New York City

## Abstract

**Introduction:**

We evaluated racial/ethnic differences in the receipt of naloxone distributed by opioid overdose prevention programs (OOPPs) in New York City (NYC).

**Methods:**

We used naloxone recipient racial/ethnic data collected by OOPPs from April 2018 to March 2019. We aggregated quarterly neighborhood-specific rates of naloxone receipt and other covariates to 42 NYC neighborhoods. We used a multilevel negative binomial regression model to assess the relationship between neighborhood-specific naloxone receipt rates and race/ethnicity. Race/ethnicity was stratified into four mutually exclusive groups: Latino, non-Latino Black, non-Latino White and non-Latino Other. We also conducted racial/ethnic-specific geospatial analyses to assess whether there was within-group geographic variation in naloxone receipt rates for each racial/ethnic group.

**Results:**

Non-Latino Black residents had the highest median quarterly naloxone receipt rate of 41.8 per 100,000 residents, followed by Latino residents (22.0 per 100,000), non-Latino White (13.6 per 100,000) and non-Latino Other residents (13.3 per 100,000). In our multivariable analysis, compared with non-Latino White residents, non-Latino Black residents had a significantly higher receipt rate and non-Latino Other residents had a significantly lower receipt rate. In the geospatial analyses, both Latino and non-Latino Black residents had the most within-group geographic variation in naloxone receipt rates compared to non-Latino White and Other residents.

**Conclusions:**

This study found significant racial/ethnic differences in naloxone receipt from NYC OOPPs. We observed substantial variation in naloxone receipt for non-Latino Black and Latino residents across neighborhoods, indicating relatively poorer access in some neighborhoods and opportunities for new approaches to address geographic and structural barriers in these locations.

## Introduction

In 2021 overdose deaths in New York City (NYC) were at their highest rate since reporting began in 2000, with fentanyl present in approximately 80% of those deaths (1). Opioid-related overdose death rates have increased significantly among non-Latino Black and Latino adults nationally and in NYC since the early 2010s. Racial/ethnic, economic, and place-based disparities in opioid overdose deaths increased as fentanyl emerged in the illicit drug market in 2016 (2–7).

Naloxone, a highly effective opioid reverse agent, is available through pharmacies and community-based distribution by opioid overdose prevention programs (OOPPs). Inadequately stocked pharmacies and pharmacy deserts in racial minority and low-income communities, especially in metropolitan areas, have led to substantial racial/ethnic inequalities in pharmacy-based naloxone access (8–10). The role of OOPPs is to promote the distribution of naloxone and make it more equitably accessible for people at risk of opioid overdose (11–13). Studies have found that naloxone distributed from OOPPs reaches neighborhoods that pharmacy-distributed naloxone does not (e.g., urban, low-income, predominately minority neighborhoods with high opioid-related overdose mortality) and increases the likelihood of overdose reversal occurring within these communities (9, 13).

In NYC, OOPPs are any program registered with New York State as a source of naloxone distribution and training, which include a range of community-based organizations, healthcare facilities, syringe service programs, and other organizations. Compared to OOPPs, pharmacies play a smaller role in naloxone distribution in NYC (14). In 2018, NYC began collecting individual-level data to understand who receives naloxone kits from OOPPs and where kits are received (15). In this study, we used data collected from OOPPs to evaluate neighborhood-level differences in the rate of community-based naloxone receipt across racial/ethnic groups in NYC. Using neighborhood-level data, our study evaluated: a) the effectiveness of OOPPs at reaching racial/ethnic minorities in a city whose strategy prioritizes OOPPbased naloxone distribution; b) whether there are differences in naloxone access in specific neighborhoods within racial/ethnic groups.

## Methods

We used data collected by the Bureau of Alcohol and Drug Use Prevention, Care and Treatment (BADUPCT) of the NYC Department of Health and Mental Hygiene (DOHMH). Individual-level naloxone recipient data was collected from 170 OOPPs using a standardized naloxone recipient form. The 170 OOPPs comprised syringe service programs (*n* = 14), correctional health services (*n* = 8), shelters (*n* = 7), drug treatment programs (*n* = 50), healthcare facilities (*n* = 48), substance use-related community-based organizations (*n* = 7), drug treatment programs (n = 50), DOHMH (n = 5), multi-component priority programs (n = 14) and others (n = 17). Data were collected between April 1, 2018, and March 31, 2019. The quarterly counts of naloxone kits received by NYC residents from OOPPs were stratified by the naloxone recipients’ neighborhood of residence designated by United Hospital Fund (UHF) neighborhoods, racial/ethnic group (16). UHF neighborhood borders are contiguous with ZIP Codes, allowing us to assign ZIP Code-level residence data to each neighborhood without overlaps. The racial/ethnic groups included in the OOPP dataset were mutually exclusive and defined as: (1) Latino/Hispanic of any race, (2) non-Latino Black, (3) non-Latino White, and (4) non-Latino Other. The non-Latino Other category included: Asian, American Indian/Alaska Native, Pacific Islander/Native Hawaiian, two or more races, other, and don’t know.

We obtained annual, neighborhood-level counts of all-type overdose deaths and opioid-related overdose deaths from the DOHMH’s Bureau of Vital Statistics and the Offi ce of the Chief Medical Examiner. However, the number of all-type and opioid-related overdose deaths were not stratified by racial/ethnic group or quarter due to data suppression guidelines.

We obtained other neighborhood-level characteristics from the United States Census American Community Survey (ACS), including the number of residents who identify as either non-Latino Black, non-Latino White, Latino or non-Latino Other, and the percentages of residents in poverty and residents with a Bachelor’s degree or higher in 2018 (US Census Bureau and American Community Survey, 2020).

Neighborhood-level characteristics were created by aggregating characteristics from ZIP codes to UHF neighborhoods. Incarceration rates for different neighborhoods, defined as the rate of current imprisonment among people who identified a specific neighborhood as their resident neighborhood at intake, were calculated using methodology from the Prison Policy Initiative (18).

We conducted a multilevel negative binomial regression model nested by UHF neighborhoods to assess the difference in naloxone receipt rates across racial/ethnic categories. The outcome variable, quarterly naloxone receipt rate in a neighborhood, was defined as the number of naloxone kits received by individuals in each racial/ethnic group according to neighborhood and quarter, with racial/ethnic stratified population sizes defined as the offset. The independent variable of interest was categorical, representing the four mutually exclusive racial/ethnic groups noted above. Other covariates included in the multivariate models were neighborhood-level annual opioid-related overdose death rate, incarceration rate, percentage of residents in poverty, and the percentage of residents over 25 years old with a Bachelor’s degree or higher. We also conducted a sensitivity analysis where we replaced the opioid-related overdose death rate with the all-type overdose death rate in the model. Given the similarity in results, the latter results are not shown.

Lastly, we used the Getis-Ord Gi* statistic for neighborhood-level geospatial analysis. Using the Gi* statistic and z-scores, we identified geospatial clustering of racial/ethnic-specific naloxone receipt rates. Hot spots were clusters of ≥ 2 adjacent UHF neighborhoods with significantly higher naloxone receipt rates than the expected rate. Cold spots were clusters of ≥ 2 adjacent UHF neighborhoods with statistically significantly lower naloxone receipt rates than the expected. We performed separate geospatial analyses for each racial/ethnic group to evaluate within-group variation in the distribution of naloxone receipt rate across neighborhoods in NYC. This analysis used R version 1.0.143, SAS version 9.4, and ArcGIS version 10. The Brown University School of Public Health and NYC DOHMH Institutional Review Boards approved and considered this study exempt.

## RESULTS

Across the 42 neighborhoods, 79,555 naloxone kits were distributed between April 2018 and March 2019. Of those kits, 28,034 were distributed to non-Latino Black residents, 27,343 to Latino residents, 15,898 to non-Latino White residents, and 8,280 to non-Latino Other residents. The median quarterly naloxone receipt rate for Black residents was 41.8 per 100,000, and for Latino residents was 22.0 per 100,000. White and Other residents had comparatively lower naloxone receipt rates across quarters of 13.6 and 13.3 per 100,000, respectively.

In the bivariate multilevel regression models (Table 1), we found that Black residents received naloxone at a significantly higher rate than White residents (Rate Ratio [RR]: 1.69, 95% CI: 1.10, 2.60). Latino and Non-Latino Other residents had lower rates of receiving naloxone than White residents at 0.90 (95% CI: 0.63, 1.30) and 0.80 (95% CI: 0.70, 0.91), respectively. When we controlled for neighborhood opioid-related overdose mortality and sociodemographic characteristics, Black residents continued to have the highest naloxone receipt rate across all racial/ethnic groups (adjusted RR [aRR]:2.10 (95% CI = 1.54–2.85 compared to White residents). Latino residents had a non-significantly higher rate (aRR = 1.11, (95%CI = 0.85–1.43), compared with White residents, whereas non-Latino Other residents had a significantly lower rate (aRR = 0.79, (95%CI = 0.67–0.92).

**Figure 1:**
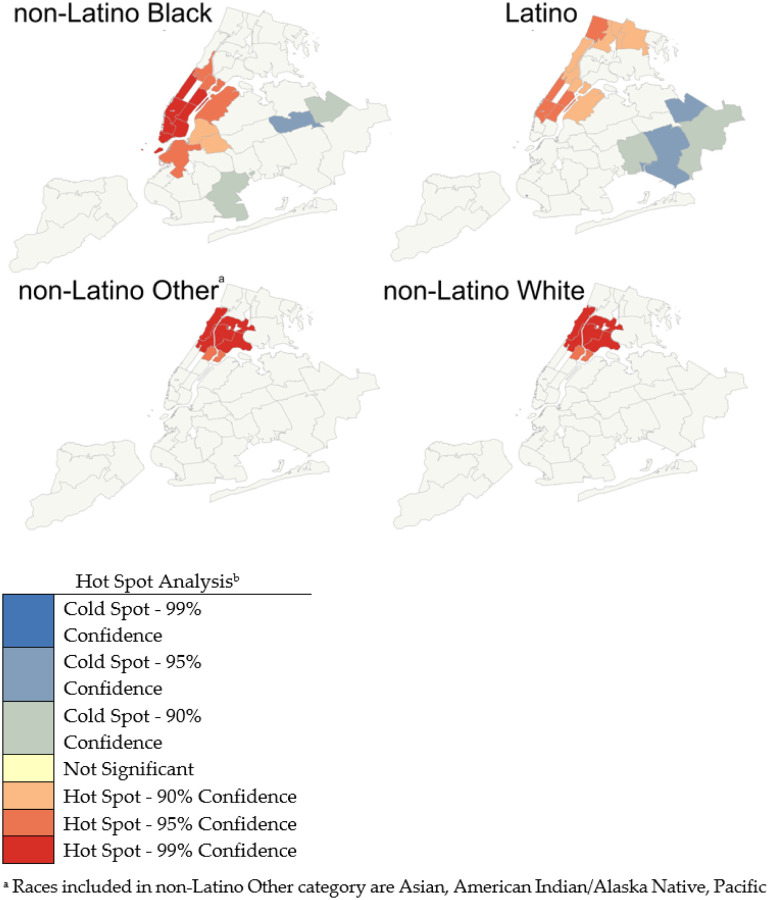
Racial/ethnic-specific hot spot analyses of the OOPP-distributed naloxone receipt rate in NYC, 2018–2019

In the geospatial analysis (Fig. 1), hot spots, where a cluster of UHF neighborhoods had substantially higher than expected naloxone receipt rates, were found in NYC’s northern and northwestern neighborhoods among all racial/ethnic groups. Non-Latino Black and Latino residents had the highest within-group variation across all neighborhoods, which was observed as having a greater number of both hot and cold spots. Specifically, cold spots were only found for the non-Latino Black and Latino groups, and these cold spots were located in the southern and southeastern areas of NYC.

## Discussion

Our study found differences in naloxone receipt between and within racial/ethnic groups in NYC neighborhoods. Non-Latino Black and Latino residents had higher rates of naloxone receipt than non-Latino White residents. In the geospatial analysis, we identified neighborhoods where naloxone was distributed at higher and lower than expected rates within each racial/ethnic group. We found hot spots in each of the racial/ethnic group analyses; however, cold spots were found in some neighborhoods in the southern and southeastern areas of NYC for non-Latino Black and Latino residents only.

Previous studies conducted in Philadelphia, Rhode Island, and Massachusetts have found similar results in which community-based naloxone was distributed at higher rates to neighborhoods with over 50% of residents identifying as Black (13, 19). However, different studies conducted elsewhere have found that predominately Black neighborhoods received naloxone at a lower rate than predominantly White neighborhoods (9, 20–22). One possible explanation for these diverging results is that the US regulation and management of naloxone distribution is local. Whether these observed differences are due to differences in rates of drug use or overdose risk or approaches to filling gaps in naloxone distribution requires further study.

When we conducted the race/ethnicity-specific geospatial analysis, we only found cold spots in the non-Latino Black and Latino analyses. Most cold spots were found in neighborhoods in Queens, which, compared to other NYC boroughs, has the highest concentration of foreign-born residents (47%) (US Census Bureau and American Community Survey, 2020). Prior studies have found foreign-born Queens residents have low healthcare utilization compared to non-immigrant residents due to language barriers and inability to afford healthcare, limited to no access to public transportation and fears about documentation status (23–25). The same structural barriers that are preventing this population from accessing healthcare may also explain why these neighborhoods are cold spots and have lower than average naloxone distribution rates among non-Latino Black and Latino residents. In addition, there is a lack of syringe service programs in NYC located in Queens (26), indicating that inequitable distribution of harm reduction programs across NYC may be disproportionality impacting non-Latino Black and Latino Queens residents.

To increase access, naloxone distribution by OOPPs should be expanded in these neighborhoods to the extent feasible and/or resources provided to expand naloxone access by other means that address distance and structural barriers. These activities should be implemented alongside additional efforts to educate foreign-born residents about the resources available when seeking social and healthcare services, reduce language and cultural barriers, and eliminate US Immigration and Customs Enforcement (ICE) enforcement in spaces where residents receive naloxone to minimize documentation status fears (25).

Our study is not without limitations. Small event counts by neighborhood meant that we: a) were only able to include overall fatal overdose rates (as opposed to race/ethnicity-specific rates) at the neighborhood level as a covariate in our study, which may have led to under- or over-estimation of the neighborhood-level overdose burden within each of the racial/ethnic groups; b) implemented larger time periods (quarters) within a one year study period which may have masked temporal trends in naloxone receipt over time; and c) collapsed multiple racial/ethnic groups into one non-Latino Other category. The results indicating that those in the non-Latino Other group received less naloxone from OOPPs than other racial/ethnic groups suggest the importance of future work to better understand naloxone receipt barriers and facilitators for the different members of this group.

## Conclusion

Our study identified racial/ethnic differences in naloxone receipt in NYC. We found that non-Latino Black residents had a higher rate of naloxone receipt from OOPPs than non-Latino White residents. However, we also identified neighborhoods with within-group differences in naloxone receipt from OOPPs among non-Latino Black and Latino residents. The next steps in this line of inquiry are to identify opportunities for naloxone distribution approaches that could assess and then address reasons for geographic and population variation in naloxone receipt and the geographic and structural barriers contributing to this.

## Figures and Tables

**Table 1:** Negative binomial regression models of the association between racial/ethnic and neighborhood-level OOPP-distributed naloxone receipt rate

	Unadjusted Rate Ratio (95% CI)	Adjusted^c^ Rate Ratio (95% CI)
Racial/Ethnic Category^a^		
Latino	0.90 (0.62, 1.30)	1.11 (0.85, 1.43)
non-Latino Black	**1.69 (1.10, 2.60)**	**2.10 (1.54, 2.85)**
non-Latino Other^b^	**0.80 (0.70, 0.91)**	**0.79 (0.67, 0.92)**
non-Latino White	ref	ref

a The racial/ethnic of people who received naloxone. White, non-Latino is the reference group

b Races included in non-Latino Other category are Asian, American Indian/Alaska Native, Pacific Islander/Native Hawaiian, two or more races, Other, and Don’t Know

c Model was adjusted by opioid-related overdose death rate, % of residents in poverty, % of residents over the age of 25 with a Bachelor’s degree or higher and incarceration rate.

## Abbreviations

OOPPs: opioid overdose prevention programs
NYC: New York City
UHF: United Hospital Fund
BADUPCT: the Bureau of Alcohol and Drug Use Prevention, Care and Treatment
DOHMH: Department of Health and Mental Hygiene
ACS: American Community Survey
